# Sensitivity of synovial fluid analysis in hematogenous native joint septic arthritis – overall accuracy and pathogen-specific differences

**DOI:** 10.1038/s41598-026-75208-6

**Published:** 2026-10-16

**Authors:** Hanna Alexandersson, Jonatan Tillander, Mats Dehlin, Tao Jin

**Affiliations:** 1 Department of Rheumatology and Inflammation Research, Institute of Medicine, Sahlgrenska Academy, University of Gothenburg, Guldhedsgatan 10A, Gothenburg, 41346 Sweden; 2 Department of Rheumatology, Sahlgrenska University Hospital, Gothenburg, Sweden; 3 Department of Infectious Diseases, Sahlgrenska University Hospital, Gothenburg, Sweden; 4 Department of Infectious Diseases, Institute of Biomedicine, University of Gothenburg, Gothenburg, Sweden

**Keywords:** Arthritis infectious, Synovial fluid, *Staphylococcus aureus*, Native joint infection, Diagnostic sensitivity, Diseases, Immunology, Medical research, Microbiology, Rheumatology

## Abstract

This study aimed to evaluate the sensitivity of synovial fluid (SF) white blood cell count (SF-WBC), SF polymorphonuclear neutrophil percentage (PMN%), and the SF/serum glucose ratio in a study population with a definitive diagnosis of hematogenous native joint septic arthritis (SA) and explore factors influencing their performance, including the causative pathogen. We conducted a retrospective register and medical record study of 57 patients with culture-verified hematogenous native joint SA identified through ICD-codes between 2016 and 2019 in western Sweden. All patients included had documented SF-WBC analysis. Clinical data and SF analyses were compared between patients above and below cutoffs for SF-WBC (50,000 cells/mm^3^) and SF/serum glucose ratio of 0.5. SF findings were also compared between *S. aureus* and other pathogens. The sensitivity of SF-WBC ≥ 50,000 cells/mm^3^ was 51%, SF/serum glucose ratio < 0.5 was 57% and PMN% ≥ 70 was 95%. Patients with SF-WBC ≥ 50,000 cells/mm^3^ were significantly older. SA caused by *S. aureus* were associated with lower PMN% and higher SF/serum glucose ratio compared to other organisms. While PMN ≥ 70% had a high sensitivity for native joint SA, the conventional cutoffs for SF-WBC and SF/serum glucose ratio showed poor sensitivity. SA caused by *S. aureus* may be associated with a less pronounced inflammatory response compared with other pathogens, a finding that warrants further investigation.

**Supplementary Information:**

The online version contains supplementary material available at https://doi.org/10.1038/s41598-026-75208-6.

## Introduction

The diagnosis of septic arthritis (SA) is clinically challenging, yet it is crucial to establish promptly due to the significant risk of adverse outcomes. Approximately 40–50% of patients experience persistent pain or permanent disability following SA^1,2^ and the reported in hospital or 30-day mortality rate ranges from 5 to 15%^3–5^. Furthermore, the incidence of SA is potentially increasing^5–7^ with particularly high incidence among the elderly^6^, a growing subpopulation in high-income countries.

The gold standard for diagnosing SA is synovial fluid (SF) culture, but unfortunately only approximately 50–70%^7,8^ of cases are culture positive. Additionally, conventional culturing is time-consuming. Studies have examined whether broad range 16 S rRNA polymerase chain reaction (PCR) assays on SF could allow for a more rapid or accurate diagnosis of SA^4^. The findings indicated that PCR did not offer any advantage over microbial culture in joint infection caused by *Staphylococcus aureus (S. aureus)* or streptococci, the most common pathogens in SA^4^. However, a study from 2019 by Sigmund et al.^9^ concluded that the automated multiplex PCR showed at least a similar performance to the SF culture in diagnosing SA but had a shorter turnaround time within five hours. In Sweden, PCR is not routinely used for diagnosing SA but may be used in cases where antibiotics were given before culture or in specific cases such as suspected *Borrelia* infection.

There are several SF-based parameters that can help to discriminate between inflammatory and infectious arthritis. The three most commonly used SF-analysis are SF-White Blood Cell (SF-WBC) count, serum/SF-glucose ratio and polymorphonuclear percentage (PMN%)^10^. A classification of SF established by Ropes and Bauer from the 1950s is still used in today’s practice. They suggested the following cutoffs for infectious arthritis: SF-WBC count ≥ 50,000 cells/mm^3^, SF/serum-glucose ratio < 0.5 and PMN% ≥90^11^. Numerous cohort studies describe the clinical features and laboratory tests for a facilitated diagnosis of SA^4,10,12,13^, and multiple studies have questioned today’s reliability of the cutoffs for distinguishing inflammatory from infectious arthritis defined by Ropes and Bauer^10,14^. Some clinical guidelines have proposed lowering the commonly used SF-WBC cutoff from ≥ 50,000 cells/mm^3^ to ≥ 20,000 cells/mm^3^^15^. Nevertheless, the threshold of 50,000 cells/mm^3^ remains the most widely reported^4,8^ and clinically applied threshold, particularly in large parts of Europe^4^. The serum/SF-glucose ratio is another widely used synovial laboratory test in suspected SA^4,10^. The classical diagnostic cutoff is a ratio of < 0.5; although some clinicians instead rely on the level of absolute SF-glucose. For PMN% in the diagnosis of SA, the cutoff is less defined, with reported thresholds ranging from 70 to 90%^10^. These cutoffs apply to native joints, but in periprosthetic joint infection (PJI) for example, the cutoff for SF-WBC count is significantly lower with threshold of > 3,000 cells/mm^3^ and PMN% >80^16,17^.

The primary objective of this research was to analyse the SF values in patients with a culture-verified hematogenous native joint SA in order to determine if the conventional cut-offs for SF-analysis (SF-WBC, serum/SF-glucose ratio and PMN%) are reliable. The secondary objective was to explore potential factors influencing these three SF-analysis tests in the context of acute SA including the causative pathogen. We hypothesized that the conventional cutoffs have limited sensitivity in diagnosing native joint SA. We further hypothesized that SF inflammatory responses vary according to the causative pathogen.

## Materials and methods

### Data sources

#### Data collection from the Western Swedish health care register

In Sweden, all inhabitants have a personal identification number (PIN) that is used to register their health care visits. Since 1997, all primary and secondary diagnoses are registered according to the Swedish version of the International Classification of Disease version 10 (ICD-10). In the present study, the Western Swedish Health Care Register (VEGA) was used to collect information (PIN, date of diagnosis, and the diagnosing hospital) on adults (aged 18 and above) with the ICD-10 codes for SA (M00.0-M00.9X/M01.0-M01.1X) between January 2016 and December 2019. Patients from Norra Älvsborgs länssjukhus (NÄL)-Uddevalla Hospital Group (NU), a county hospital and Sahlgrenska University Hospital (SU) in Gothenburg were included. This yielded 475 patients whose medical records were reviewed. We also collected information from VEGA about selected comorbidities and risk factors present before and/or on the day when the patient was admitted to the hospital due to SA. The selected comorbidities and risk factors were established risk factors for septic arthritis^4^ with the ICD-10-codes: kidney failure (N17-N19), diabetes mellitus (E10-E14), dialysis (Z491, Z492, Z94, Z966), alcoholic liver disease (K70), rheumatoid arthritis (M05-M06), juvenile arthritis (M08-M09) and gout (M10).

### Data collection from medical records

The medical records of the 475 patients were reviewed by one of the authors (H.A), a rheumatology resident. In cases of uncertainty, a senior specialist in rheumatology (T.J) and a senior specialist in infectious diseases (J.T) were consulted. Collected data included:

- Demographics: age, sex, length of hospital stay (LOS), number of affected joints and site of SA and mortality during hospitalization and within 30 days after discharge from the hospital.
- Route of infection and previous surgery or intra-articular procedures in the affected joint within 3 months before admission.
- Presence of a prosthesis or synthetic material in the affected joint.
- Information about current immunosuppressive medication during the hospital admission period and the presence of an active skin ulcer at the admission was registered.
- Parameters: The body temperature measured either at the emergency ward or within the first 24 h after admission, along with CRP measured at admission. SF-WBC count, SF-glucose and blood glucose as well as SF-neutrophils (to calculate PMN%). In all included cases, synovial fluid was obtained by joint aspiration before any arthroscopic washout or surgical intervention. Data on immune status, such as Mannose-Binding Lectin levels, were not available.
- Time to aspiration: By carefully reviewing the medical record the time span between the onset of symptoms (pain, swelling or fever of unknown origin) and the joint aspiration was determined and then referred to as “time to aspiration”.
- Culture results: from SF, synovial tissue when performed and blood were collected, and antimicrobial resistance patterns were registered. The culture results were validated by thorough review of medical records to ensure their relevance to the diagnosis of SA. Only patients with isolated organisms from serum or synovia were included. In cases with negative synovial culture but positive blood culture (*n* = 4), the medical records were thoroughly examined to confirm diagnosis. When PCR assay of the SF contributed to the diagnosis of SA, this was also recorded.

### Study population

**Fig. 1:**
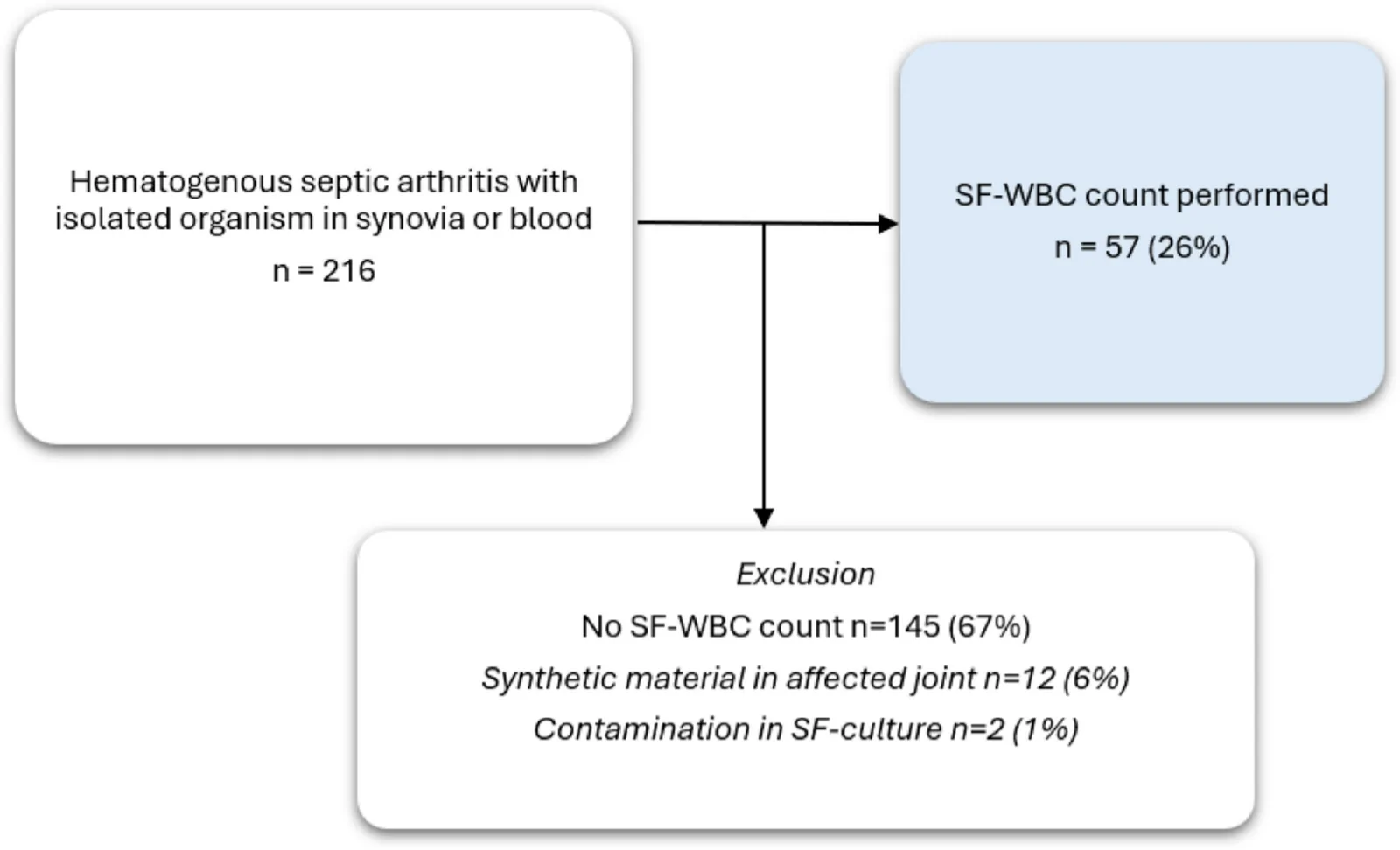
Patient Selection Flowchart. The study population (*n* = 57) consists of patients with culture-verified SA with an analysis of SF-WBC count. Fifty-three (93%) of the patients had a positive culture from the joint whereas four patients (7%) had a positive blood culture alone. In 30 patients (53%) the same organism was isolated from both the joint aspirate and the blood culture.

Patient selection is outlined in Fig. 1. The inclusion criteria were adult patients with ICD-10-codes for SA who were admitted to SU and NU between 1:st of January 2016 and 31:st of December 2019. The exclusion criteria were absence of an isolated organism, a direct inoculation route of infection (defined as surgery or intra-articular procedure in the affected joint within 3 months or a skin ulcer following joint trauma or animal bite within 10 centimetres of the affected joint), missing SF-WBC count, and presence of synthetic material in affected joint. Thus, the final study population consisted exclusively of patients with a spontaneous hematogenous septic arthritis of native joints.

Since our aim was to describe and analyse the classic tests of the SF in patients with a definitive culture-verified diagnosis of SA, the remaining patients were carefully examined again through the medical record. During this process, two patients with coagulase-negative staphylococci growth in SF were identified, but review of the medical record indicated that those findings most likely represented contamination rather than true infection. Consequently, these two patients were excluded.

Patients with synthetic material in the joint (including prosthetic joint infection (PJI)) have a different diagnostic cutoff for the SF-WBC^16^ and are often managed differently. Therefore, only cases of native joint infection were included in the final analysis.

### Statistical analysis

Descriptive statistics were used to summarize the demographics and characteristics of the study population. For subgroup comparisons of patients with SF-WBC < 50,000 cells/mm^3^ versus ≥ 50,000 cells/mm^3^ and SF/serum glucose ratio < 0.5 versus ≥ 0.5 respectively the Student´s t-test, the Chi-square test or Fisher´s exact test was used when appropriate. Statistical analyses were performed using GraphPad Prism 10 or SPSS Statistics for Windows, Version 28 (released 2021; IBM Corp., New York, NY, USA) and statistical significance was set at *p* < 0.05. This retrospective study was approved by the Swedish Ethical Review Authority and conducted in accordance with the Declaration of Helsinki. The requirement for informed consent was waived by the Swedish Ethical Review Authority due to the retrospective design, the large study population, and the use of existing registry and medical record data, with no risk of individual identification.

## Results

**Fig. 2:**
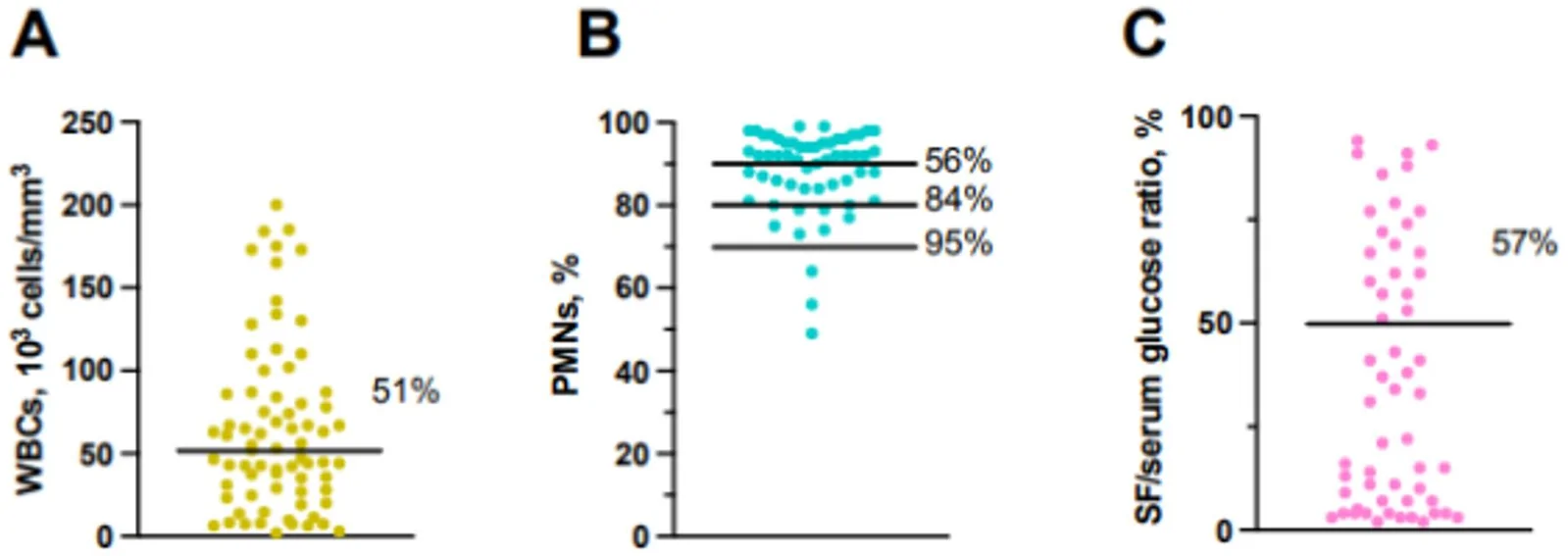
Percent of the study population of hematogenous native joint infections fulfilling the traditional cutoffs for SF-WBC count ≥ 50,000 cells/mm^3^ (51%), %PMNs ≥ 70 (95%), ≥ 80 (84%) and ≥ 90 (56%). In 57% of the cases, the SF/serum glucose ratio was < 0.5.

The sensitivity of the 50,000 cells/mm^3^ synovial WBC cutoff was 51% (29 of 57 patients). Of 55 patients, 95% (52 patients) had PMN% ≥70, 84% (46 patients) had PMN% ≥80, and 56% (31 patients) had PMN% ≥90. The sensitivity of the SF/serum glucose ratio < 0.5 cutoff was 57% (24 of 42 patients) (Fig. 2).

Total and subgroup demographics stratified by SF-WBC count (< 50,000 vs. ≥50,000 cells/mm^3^) and by SF/serum glucose ratio (< 0.5 vs. ≥ 0.5) are shown in Table 1. Since PMN% ≥70 demonstrated a high sensitivity, we did not further analyse differences in this subgroup. The detailed description of patients with SF-WBC below 10,000 cells/mm^3^ is shown in supplementary Table 1.

**Table 1:** Demographics of study population and comparison between subgroups over or below SF-WBC count 50,000 cells/mm^3^ and over or below SF/serum-glucose ratio 0.5.

Parameter	Total *n* = 57	SF-WBC < 50 000 cells/mm^3^ *N* = 28	SF-WBC ≥ 50 000 cells/mm^3^ *N* = 29	SF/serum glucose ratio ≥ 0.5 *n* = 18	SF/serum glucose ratio < 0.5 *n* = 24
Sex, N (%)	F: 24 (42)	F: 14 (50)	F: 10 (34)	F: 8 (44)	F: 10 (42)
	M: 33 (58)	M: 14 (50)	M: 19 (66)	M: 10 (56)	M: 14 (58)
Age years, median (range)	72 (19–96)	64 (19–96)	77 (30–91)	66 (22–96)	74 (28–91)
Age years, mean (SD)	69 (18)	63 (20)	74 (14)*	67 (19)	71 (16)
Number of joints involved					
Monoarthritis, N (%)	54 (95)	27 (96)	27 (93)	17 (94)	23 (96)
Two joints, N (%)	2 (3.5)	1 (4)	1 (3.4)	0 (0)	1 (4)
Three or more joints, N (%)	1 (1.8)	0 (0)	1 (3.4)	1 (6)	0 (0)
Infected joint, N (%)					
Knee	41 (72)	19 (68)	22 (76)	16 (89)	19 (79)
Glenohumeral	4 (7.0)	1 (3.6)	3 (10)	0 (0)	2 (8.3)
Ankle	4 (7.0)	3 (11)	1 (3.4)	0 (0)	1 (4.2)
Hip	2 (3.5)	2 (7.1)	0 (0)	1 (5.6)	1 (4.2)
Foot	1 (1.8)	1 (3.6)	0 (0)	0 (0)	0 (0)
Wrist	1 (1.8)	0 (0)	1 (3.4)	0 (0)	0 (0)
Elbow	1 (1.8)	1 (3.6)	0 (0)	0 (0)	0 (0)
Polyarticular^a^	3 (5.3)	1 (3.6)	2 (6.9)	1 (5.6)	1 (4.2)
Time to aspiration, hours, median (range)^b^	96 (10–816)	168 (16–720)	72 (10–816)	168 (16–720)	60 (10–816)
Time to aspiration, hours, mean (SD)	161 (174)	182 (163)	142 (183)	182 (164)	148 (194)
Fever ≥ 37.5 Celsius, N (%)^c^	31 (54)	16 (57)	15 (52)	9 (50)	14 (58)
P-WBC^b^ cells/mm^3^ mean (SD)	15 488 (21 420)	12 919 (6 998)	17 879 (29 043)	13 729 (8 583)	11 954 (5 623)
CRP, mg/L mean (SD)^d^	211 (121)	222 (130)	200 (112)	209 (97)	206 (130)
Culture site N (%)					
Synovial fluid or tissue	53 (93)	24 (86)	29 (100)	15 (83)	24 (100)
Blood	4 (7)	4 (14)	0 (0)	3 (17)	0 (0)
	*N* = 48	*N* = 22	*N* = 26	*N* = 17	*N* = 22
Crystals in synovial fluid, N (%)	14 (29)	5 (23)	9 (33)	4 (24)	6 (27)
MSU	6 (12)	1 (4.5)	5 (19)	2 (12)	3 (13.5)
CPPD	7 (15)	4 (18)	3 (11)	2 (12)	3 (13.5)
MSU and CPPD	1 (2.0)	0 (0)	1 (3.7)	0 (0)	0 (0)
Joint disease, N (%)	18 (32)	10 (36)	8 (28)	7 (39)	6 (25)
Rheumatoid arthritis	5 (8.8)	4 (14)	1 (3.4)	1 (5.6)	3 (13)
Juvenile arthritis	1 (1.8)	1 (3.6)	0 (0)	1 (5.6)	0 (0)
Gout	12 (21)	5 (18)	7 (24)	5 (28)	3 (13)
Risk factors, N (%)	37 (65)	14 (50)	23 (79)	12 (67)	14 (58)
Kidney failure	14 (25)	7 (25)	7 (24)	4 (22)	6 (25)
Diabetes mellitus	10 (18)	3 (11)	7 (24)	5 (28)	4 (17)
Dialysis	4 (7.0)	0 (0)	4 (14)	2 (11)	1 (4.2)
Alcoholic liver disease	2 (3.5)	1 (3.6)	1 (3.4)	1 (5.6)	0 (0)
Skin ulcer	7 (12)	3 (11)	4 (14)	0 (0)	3 (12)
Immunosuppression, N (%)	11 (19)	5 (18)	6 (21)	4 (22)	4 (17)
Infliximab + methotrexate	1 (1.8)	1 (3.6)	0 (0)	0 (0)	1 (4.2)
Betamethasone 4 mg/day	1 (1.8)	1 (3.6)	0 (0)	1 (5.6)	0 (0)
Prednisolone^e^	7 (12)	2^g^ (7.1)	5^h^ (17)	3^i^ (17)	3^j^ (13)
Chlorambucil + prednisolone 5 mg/day	1 (1.8)	1 (3.6)	0 (0)	0 (0)	0 (0)
Cetuximab	1 (1.8)	0 (0)	1 (3.4)	0 (0)	0 (0)
Length of hospital stay (LOS), days, median (range)	19 (2–78)	19 (3–48)	20 (2–78)	24 (17)	24 (11)
Mortality					
During hospitalization, N (%)	3 (5.3)	1 (3.6)	2 (6.9)	1 (5.6)	2 (8.3)
Within 30 days^f^, N (%)	3 (5.3)	1 (3.6)	2 (6.9)	1 (5.6)	2 (8.3)

MSU: Monosodium urate. CPPD: Calcium pyrophosphate dihydrate.

All differences between the group with SF-WBC over and under 50 and SF/serum glucose ratio over or below 0.5 have been statistically analysed. Any significant differences found are marked with *. Percentages may not add up to 100% due to rounding.

^a^Two patients with affected: knee and glenohumeral joint. One patient with affected: knee bilateral, ankle, wrist, and sternoclavicular joint.

^b^Time from onset of symptoms to aspiration of the joint.

^c^At the emergency ward or within the first 24 h after admission.

^d^At admission.

^e^Prednisolone: dose 5–10 mg/day *n* = 3, ≥ 10–30 mg/day *n* = 4.

^f^After discharge from hospital.

^g^Prednisolone 5–10 mg/day. ^h^Prednisolone 5–10 mg/day *n* = 1, ≥ 10–30 mg/day *n* = 4. ^i^Prednisolone 5–10 mg/day *n* = 1, ≥ 10–30 mg/day *n* = 2.

^j^Prednisolone 5–10 mg/day *n* = 1, ≥ 10–30 mg/day *n* = 2.

*p-value 0.02 evaluated with Independent Samples t-test.

### Patient characteristics

The demographics of the total study population are detailed in Table 1. Of the entire cohort, 33 patients (58%) were men, and the median age was 72 years (range 19–96). The most common joint location was the knee (72%) followed by the glenohumeral (7.0%), ankle (7.0%) and hip (3.5%). The median time to aspiration was 96 h (4 days). Fever (≥ 37.5 Celsius) during the first 24 h after arrival at the emergency ward was present in 31 patients (54%), the mean CRP was 211 mg/L ± 121 mg/L, with CRP ≥ 30 mg/L in 55 (96%) of the cases. Crystals in SF were present in 14 (29%) patients, with monosodium urate (MSU) crystals and calcium pyrophosphate dihydrate (CPPD) found in 12% and 15% of cases, respectively (Table 1). Inflammatory joint disease was prevalent in 18 (32%) of the patients and 12 of the patients (21%) had a previous diagnosis of gout. The median LOS was 19 days (range 2–78 days), and the mortality rate within 30 days after admission was 5.3% (3 patients).

### Comparison between subgroups

Male sex predominated in all subgroups except the group with SF-WBC < 50,000 cells/mm^3^, although this difference was not statistically significant. No difference was found in the joint distribution or in the frequency of crystals in SF (Table 1).

Age was the only variable that differed significantly between the subgroups, with patients in the high SF-WBC count group being older (*p* = 0.02). The time to aspiration of the joint as well as, the peripheral concurrent WBC-levels, immunosuppressant use, and the presence of crystals in SF did not explain the observed difference (Table 1). Since there was a higher age observed in the high SF-WBC group, we further analysed whether the sensitivity of SF-WBC cutoff of > 50,000 cells/mm³ differed between younger (≤ 65 years) and older (> 65 years) patients. Interestingly, the > 50,000 cells/mm³ cutoff identified only 29.0% of younger patients compared with 65.8% of older patients (*p* < 0.01).

As microorganisms were isolated from SF alone in 23 patients (40%), from both blood and SF in 30 patients (53%), and from blood alone in 4 patients (7%), we compared SF parameters across these subgroups (Supplementary Table 2). No statistically significant differences were observed. Notably, none of the four patients with microorganisms isolated from blood alone had an SF-WBC count > 50,000 cells/mm³.

### Isolated organisms

The most common isolated organism was *S. aureus* accounting for 29 cases (51%) followed by β-hemolytic streptococci in 10 cases (18%) (Table 2). In three cases (5.3%) two different organisms were isolated in culture from SF. PCR assays attributed to the diagnosis in three cases (5%) and all of which also later had a positive culture. No methicillin-resistant *Staphylococcus aureus* (MRSA) or extended-spectrum beta-lactamase (ESBL) producing organisms were identified.

**Table 2:** Isolated organisms in 57 patients withhematogenous native joint septic arthritis.

Organism	Total *N* = 57 (%)	Isolated organism in from synovia, *N* = 53 (93%)	Isolated organism in blood alone, *N* = 4 (7.0%)
*S. aureus*	29 (51)	27 (47)^a^	2 (3.5)
β-hemolytic streptococci	10 (18)	9 (16)	1 (1.8)
Group G	4 (7.0)	4 (7.0)	0 (0)
Group A	2 (3.5)	1 (1.8)	1 (1.8)
Group B	2 (3.5)	2 (3.5)	0 (0)
Group C^b^	2 (3.5)	2 (3.5)	0 (0)
*Streptococcus pneumonia*	6 (11)	5 (8.7)	1 (1.8)
Others	5 (8.7)	5 (8.7)	0 (0)
*Citrobacter koseri*	1 (1.8)	1 (1.8)	0 (0)
*Enterococcus faecalis*	1 (1.8)	1 (1.8)	0 (0)
*Haemophilus influenzae*	1 (1.8)	1 (1.8)	0 (0)
*Serratia marcescens*	1 (1.8)	1 (1.8)	0 (0)
*Streptococcus mitis*	1 (1.8)	1 (1.8)	0 (0)
Mixed infections	3 (5.3)	3 (5.3)	0 (0)
β-hemolytic group B + *Streptococcus dysgalactiae*^b^	1 (1.8)	1 (1.8)	0 (0)
*Eschericha coli* + *Staphylococcus aureus*	1 (1.8)	1 (1.8)	0 (0)
*Staphylococcus aureus* + *Streptococcus sp*^b^	1 (1.8)	1 (1.8)	0 (0)
*Eschericha coli*	3 (5.3)	3 (5.3)	0 (0)
*Pseudomonas aeruginosa*	1 (1.8)	1 (1.8)	0 (0)

^a^Isolated *S. aureus* in both synovia (synovial fluid or synovial tissue) and blood (*n* = 17). ^b^PCR assay attributed to the diagnosis.

To assess whether the SF-analyses differed by causative organisms, *S. aureus* infections were compared with all other organisms (Table 3). There was a lower proportion of patients meeting PMN% ≥90 threshold in SA caused by *S. aureus* (*p* = 0.03), and there was a non-significant trend towards lower overall PMN% in *S. aureus* infections compared to other organisms (Table 3).

**Table 3:** Comparison between patients with *Staphylococcus aureus* as the isolated organism and patients with other isolated organisms.

	Staphylococcus aureus *N* = 31 (54)	Other isolated organism *N* = 26 (46)
Age, mean (SD)	64 (20)	73 (13)^a^
CRP mg/L, mean (SD)	192 (125)	233 (115)
	*N* = 30	*N* = 26
WBC cells/mm^3^, mean (SD)	18 680 (28,722)	11 804 (5,110)
Fever ≥ 37.5 Celcius, N (%)	18 (58)	13 (50)
	*N* = 30	*N* = 26
Time (h) from pain to puncture of the joint, mean (SD)	166 (148)	156 (202)
SF-WBC x cells/mm^3^, mean (SD)	52 (43)	67 (52)
SF-WBC ≥ 50,000 cells/mm^3^, N (%)	15 (48)	14 (54)
SF-WBC ≥ 70 cells/mm^3^, N (%)	7 (23)	9 (35)
	*N* = 30	*N* = 25
PMN%, mean (SD)	86 (10)	90 (11)
PMN% ≥70, N %	28 (93)	24 (96)
PMN% ≥80, N %	23 (77)	23 (92)
PMN% ≥90, N %	13 (43)	18 (72)^b^*
	*N* = 21	*N* = 21
Glucose ratio, mean (SD)	0.51 (0.31)	0.30 (0.30)^c^
Glucose ratio < 0.5, N %	9 (43)	15 (71)^d^

If mixed infection: if *S. aureus* was isolated, the patient was included in the group: *S. aureus*.

Mixed infection occurred in 3 patients. Continuous variables were compared using Student’s t-test, whereas categorical variables were compared using the Chi-square test, as appropriate. *p < 0.05.

^a^NS, p-value 0.058 with Independent Samples t-test.

^b^*p* = 0.033 with Chi^2^-test.

^c^NS, p-value 0.064 with Independent Samples t-test.

^d^NS, p-value 0.061 with Chi^2^-test.

**Fig. 3:**
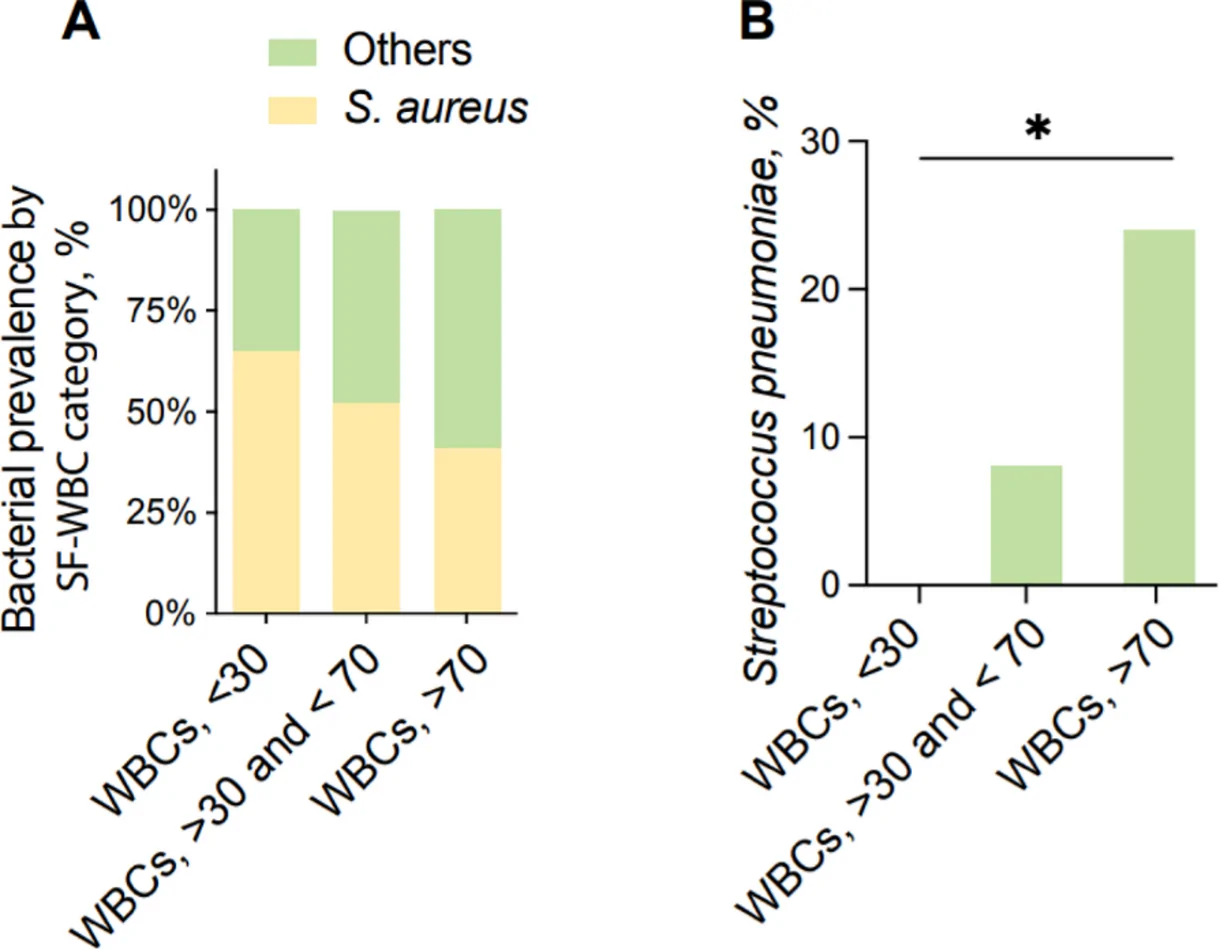
Causative organisms and the levels of SF-WBC count in three groups: < 30,000 cells/mm^3^ (low), ≥ 30,000 and < 70,000 cells/mm^3^ (medium) and ≥ 70,000 cells/mm^3^ (high). (**A**) Demonstrates *S. aureus* in relation to all other organisms (*n* = 57). (**B**) Demonstrates the distribution of patients infected with *Streptococcus pneumoniae* (*n* = 6). *p-value < 0.05 for comparison between the low and high SF-WBC groups; Fisher exact test was used.

To further analyse differences between patients infected with *S. aureus* and those infected with other causative organisms, we stratified the study population into three subgroups based on SF-WBC: < 30,000 cells/mm^3^ (low), ≥ 30,000 to < 70,000 cells/mm^3^ (medium) and ≥ 70,000 cells/mm^3^ (high) (Fig. 3).

The majority (65%) of the patients infected with *S. aureus* belonged to the low SF-WBC group, whereas infections caused by other bacteria, most patients (60%) were classified in the high SF-WBC group (Fig. 3). Further analysis of non-*S. aureus* group showed that *Streptococcus pneumoniae* frequencies increased with higher SF-WBC counts, from 0% in SF-WBCs < 30,000 group to 23% in SF-WBCs ≥ 70,000 group, although this is based on only 6 patients.

## Discussion

The most important finding of this study was that in our cohort, SF-PMN% ≥70 was the most sensitive test, whereas the sensitivity of SF-WBC count ≥ 50,000 cells/mm³ and the SF/serum glucose ratio was insufficient for reliably diagnosing hematogenous native joint SA. The present study confirms previous findings^3,10,12,14,18,19^ that commonly used cutoffs for SF analysis have insufficient sensitivity for diagnosing SA. The widely applied cutoff for SF-WBC count (≥ 50,000 cells/mm^3^) had a sensitivity between 53 and 70% from meta-analysis by Margretten et al.^10^. An important limitation of the mentioned meta-analysis was the variation in inclusion criteria and definitions of the diagnosis.

To ensure a homogenous cohort in the present study, only patients with hematogenous native joint infection and a positive culture from synovia or blood was included. Still, only 51% of the patients had SF-WBC count ≥ 50,000 cells/mm^3^. This is in line with a systematic review of 11 studies by Carpenter et al. from 2011^20^ which found a pooled sensitivity of 56% (lowest value 31%) for SF-WBC count ≥ 50,000 cells/mm^3^ in diagnosing SA.

In the current study, patients with PJI were excluded but patients receiving immunosuppressants were not. Some previous studies have suggested that leukopenia may affect the thresholds for SF-WBC count in SA but that SF-PMN% would be a more reliable analysis in those cases^21^. In our study the number of patients receiving immunosuppressants was small, and no significant difference was found between the patients with or without immunosuppressants (Table 1).

In the clinical setting, distinguishing crystal arthropathy flares from SA might be challenging. This is because the clinical presentation may overlap, crystals in synovial fluid may be present without crystal arthropathy, and SA and crystal arthropathy may co-exist^18^. In the present study 29% of the patients had crystals in SF (Table 1) despite having culture-verified SA. There are limited studies evaluating how the diagnostic criteria of SA differ between patients with and without SF crystals^13,22^. In a study by Pesante et al. with 54 patients with SA^22^, only 5 patients (9.3%) had crystals in SF. They concluded that the SF-WBC count was higher in the group with crystal arthropathy, and that SF-PMN% was less useful in the cases with co-existing crystal arthropathy. In the present study, there was no significant difference in SF-WBC count between patients with and without crystals in SF (Table 1). Unfortunately, this study lacks a control group of patients with crystal arthritis alone, which would be highly valuable to investigate in order to improve diagnostic accuracy and should be explored in future studies. Another conventional SF test used to diagnose SA is SF/serum glucose ratio (Mathews 2008). The sensitivity of the SF/serum glucose ratio < 0.5 in the present study was 57%, which is in line with previous studies^10^. The SF-analysis with the highest sensitivity in our study was SF-PMN% ≥70 with a sensitivity of 95%. However, when SF-PMN% cutoff was set at ≥ 90, as commonly reported in the literature, the sensitivity decreased to 56%. These figures are highly consistent with previous studies; for example, Varady et al.^23^ which found a sensitivity of 65% for SF-PMN% ≥ 90 in 598 patients, and Carpenter et al.^20^ found a pooled sensitivity of 60% for SF-PMN% ≥ 90 (3 studies). The meta-analysis by Margretten et al.^10^ presents a sensitivity range of 57–92% for SF-PMN% ≥90. A retrospective study by Rasmussen et al.^19^ with 40 culture-verified native joint infections demonstrated SF-PMN% to be one of the most accurate laboratory predictors of SA with a median SF-PMN of 93%, and that most patients had SF-WBC count (median 32,435 cells/mm^3^) below the 50,000 cells/mm^3^ cutoff.

The inherent trade-off between sensitivity and specificity should be considered when defining diagnostic thresholds. Rather than merely adjusting diagnostic thresholds, we suggest a multifaceted diagnostic approach when evaluating patients with acute arthritis. As no single formula or algorithm can reliably establish or exclude the diagnosis of SA, the importance of clinical judgement remains central to optimal diagnosis and care.

In this study, we focused on the SF-analysis, but also noted that the CRP was consistently high, with mean CRP 211 mg/L, and 96% of the patients had CRP ≥ 30 mg/L. This is consistent with prior studies^10,18^, which have concluded that CRP has a high sensitivity but a low specificity. Nevertheless, it is highly unlikely that a patient with CRP < 10 mg/L has SA, although this also applies to crystal arthropathy flare as well as most flares in inflammatory joint diseases. Thornton et al.^24^ showed that patients with CRP levels of 90 to 200 mg/L could have either SA or crystal arthropathy, but all of the patients with CRP > 200 mg/L had SA. Overall, there is a wide overlap in CRP levels between SA and crystal arthropathy, even though very high CRP (over 200 mg/L) increases the likelihood of SA^24^. When comparing patients with SF-WBC over or below 50,000 cells/mm^3^ or SF/serum glucose ratio over or below 0.5, the only significant difference was that the patients with higher SF-WBC were older. Although time to aspiration and comorbidities did not differ significantly, there was a trend toward a higher burden of comorbidities in the high SF-WBC group, which also had a significantly higher age – a known risk factor for SA. Other potential factors, such as immunosuppressive therapy or the presence of crystals in SF, did not differ significantly. Clinically, this may suggest that younger patients with SA more often present with lower SF-WBC counts, and in these patients the SF-PMN% and clinical evaluation are especially important.

Compared with other organisms, *S. aureus* consistently showed a non-significant trend toward lower SF-WBC. Similarly, SF-PMN% was generally lower in the *S. aureus* group compared with other organisms, with a significant difference at the ≥ 90% cutoff.

This raises the question of whether the higher SF-WBC count reported in the Ropes and Bauer cohort from the 1950s^11^ could, at least in part, be explained by differences in pathogen distribution compared to today. Unfortunately, the authors did not provide data on the frequency of specific pathogens across SF-WBC count ranges^11,25^. Of note, Howard et al.^26^ reported that the incidence of SA caused by *Haemophilus influenzae* type B markedly declined in Canada after the introduction of vaccination in 1992 illustrating that the spectrum of causative microorganisms change which likely impacts the host immune response and consequently the results of SF-analysis could also change.

To our knowledge, few studies have analysed the SF-WBC count in relation to causative pathogens^19,25^, and the sample sizes have been small. Coutlakis et al.^25^ found that 82% (14 of 17) of *S. aureus* cases had a SF-WBC count ≥ 50,000 cells/mm^3^, but 14% had SF-WBC count < 20,000 cells/mm^3^. However, direct comparison with the present study is limited due to differences in inclusion criteria. Rasmussen et al.^19^ did not stratify by pathogen, but their high proportion of *S. aureus* (67%) and low mean SF-WBC (32,435 cells/mm^3^) are consistent with our findings. Moreover, their inclusion criteria are more comparable to those used in the present study. Interestingly, in the current study 6 of 10 (60%) patients with SF-WBC count < 10,000 cells/mm^3^ were infected with *S. aureus* (Supplementary Table 1).

The association of *S. aureus* infections with a trend of lower SF-WBC counts and lower PMN% was unexpected, as *S. aureus* is typically considered a highly virulent pathogen. The reasons for this association cannot be determined from this retrospective clinical design, but we can speculate on possible mechanisms. One explanation is that *S. aureus* uses immune evasion strategies that dampen host inflammatory responses to avoid excessive immune activation that could ultimately harm themselves. For example, the chemotaxis inhibitory protein of staphylococci (CHIPS) and the staphylococcal complement inhibitor (SCIN) can effectively block chemotaxis and subsequent phagocytosis by host immune cells^27^.

Another possibility is that *S. aureus* actively kills infiltrating leukocytes through the production of toxins such as panton-valentine leukocidin^28^ and phenol-soluble modulins (PSMs)^29^. A notable example is PSMα, which potently lyses leukocytes at very low concentrations. Interestingly, a PSMα deficient *S. aureus* strain causes SA in mice with similar joint severity to its parental strain but induces far fewer kidney abscesses and less weight loss^30^. This suggests that *S. aureus* SA may initially trigger leukocyte infiltration into the joint, but toxin-mediated killing reduces the number of viable leukocytes detectable in the SF. These findings warrant further investigation, for example by comparing neutrophil chemoattractant levels such as IL-8 in SF from SA cases caused by *S. aureus* versus *S. pneumoniae*.

This study has several limitations, including its retrospective design and small sample size, largely due to our strict inclusion criteria. In particular, the parameter “time to joint aspiration” may be subject to interpretative inaccuracies, as it is sometimes challenging to determine from medical records and in clinical practice. Comparisons with other studies are further limited by differences in synovial fluid analysis methods across different countries. Furthermore, the SF-WBC counts may fall with the transport time to the laboratory, which can vary between hospitals and may affect comparability of results. Finally, owing to the lack of a control cohort comprising patients with non-infectious arthritis, the specificity of SF parameters for the diagnosis of SA could not be assessed.

However, our strategy with a strict inclusion criterion ensured that only pure SF- or blood- culture-positive hematogenous native joint SA cases were included, strengthening the robustness of our conclusions. Despite limitations, our data offer useful insights into the diagnostic challenges of SA and highlight important factors to consider in clinical evaluation.

In conclusion, only slightly more than half of patients in this study with a hematogenous native joint culture-verified SA fulfilled the conventional thresholds for SF-WBC and SF/serum glucose ratio. Of the tests evaluated, PMN% demonstrated the highest reliability. In addition, this study reveals novel findings that SA caused by *S. aureus* may be associated with a less pronounced inflammatory response compared with other pathogens, a finding that warrants further investigation.

## Supplementary Information

Below is the link to the electronic supplementary material.

Supplementary Material 1

Supplementary Material 2

Supplementary Material 3

## Data Availability

All data generated or analysed during this study are included in this published article and its supplementary information files.
