# Supplementary material for: Sensitivity of synovial fluid analysis in hematogenous native joint septic arthritis – overall accuracy and pathogen-specific differences
Source: Sci Rep. 2026 Oct 16;16:32695. doi: 10.1038/s41598-026-75208-6 (PMC13675130; doi:10.1038/s41598-026-75208-6)
Supplement: Supplementary file 1 [file 41598_2026_75208_MOESM1_ESM.docx]

**Supplementary table 1:**

| **Supplementary table 1: Description of patients with SF-WBC below 10 cells/mm^3^** | | | | | | | | | | | |
| --- | --- | --- | --- | --- | --- | --- | --- | --- | --- | --- | --- |
| **Patient number** | **Age, years** | **SF-WBC cells/mm^3^** | **PMN%** | **Glucose ratio** | **P-WBC cells/mm^3^** | **CRP mg/L** | **Time to punction, hours** | **Joint** | **Organism** | **Culture positive from Synovia (S) or Blood (B)** | **Immunosupp.** |
| 1 | 64 | 2,000 | - | 0,10 | 7,300 | 42 | 34 | hip | β-hemolytic streptococci group B | S | - |
| 2 | 82 | 3,000 | - | - | 18,600 | 97 | - | foot (MTP) | *S. aureus* | B | - |
| 3 | 65 | 6,300 | 84% | 0,74 | 15,600 | 340 | 336 | knee | *S. aureus* | S | - |
| 4 | 78 | 6,400 | 97% | 0,53 | 10,900 | 224 | 192 | knee | β-hemolytic streptococci group A | B | - |
| 5 | 56 | 7,300 | 56% | 0,04 | 17,400 | 44 | 48 | ankle | *S. aureus* | S/B | Infliximab 200 mg every 8:th week and methotrexate 15 mg per week |
| 6 | 40 | 7,400 | 73% | 0,94 | 3,800 | 150 | 216 | knee | *S. aureus* | B | - |
| 7 | 65 | 7,400 | 80% | - | 13,100 | 400 | 48 | knee | β-hemolytic streptococci group B and *Streptococcus dysgalactiae* | B | - |
| 8 | 50 | 7,800 | 77% | 0,10 | 15,400 | 384 | 192 | ankle | *S. aureus* | S/B | - |
| 10 | 86 | 9,600 | 88% | - | 14,100 | 370 | 48 | knee | *S. aureus* | S/B | - |
