# Supplementary material for: Sensitivity of synovial fluid analysis in hematogenous native joint septic arthritis – overall accuracy and pathogen-specific differences
Source: Sci Rep. 2026 Oct 16;16:32695. doi: 10.1038/s41598-026-75208-6 (PMC13675130; doi:10.1038/s41598-026-75208-6)
Supplement: Supplementary file 2 [file 41598_2026_75208_MOESM2_ESM.docx]

| **Supplementary Table 2: Comparison between subgroups due to where the organism was identified; in synovial fluid alone (SF), in synovial fluid and blood (SF + B) or only in blood (B)** | | | | |
| --- | --- | --- | --- | --- |
| **Parameter** | **Total, N=57** | **SF, N=23** | **SF + B, N=30** | **B, N=4** |
| Sex, N (%) | F: 24 (42)  M: 33 (58) | F: 10  M: 13 | F: 12  M: 18 | F: 2  M: 2 |
| Age years median (range)  Age years mean (SD) | 72 (19-96)  69 (18) | 66 (19-90)  62 (20) | 76 (50-96)  74 (14) | 70 (40-82)  66 (19) |
| SF-WBC, cells/mm^3^ mean (SD) | 59,888 (47,829) | 65,343 (48, 968) | 61,863 (47,422) | 13,700 (16,309) |
| SF-WBC > 50,000 cells/mm^3^ | 29 (51) | 13 (57) | 16 (53) | 0 (0) |
| SF/serum glucose ratio, mean (SD) | N=42 | N=18  0.37 (0.35) | N=21  0.39 (0.30) | N=3  0.68 (0.23) |
| SF-glucose ratio <0.5, N (%) | 24 (57) | 12 (67) | 12 (57) | 0 (0) |
| SF-PMN%, mean | N=55 | N=22  0.90 (0.08) | N=30  0.86 (0.11) | N=3  0.89 (0.14) |
| SF-PMN >70%, N (%) | 52 (95) | 21 (95) | 28 (93) | 3 (100) |
| SF-PMN >80%, N (%) | 46 (84) | 21 (95) | 23 (77) | 2 (67) |
| SF-PMN >90%, N (%) | 31 (56) | 12 (55) | 17 (47) | 2 (67) |
